# Enzymes and genes involved in aerobic alkane degradation

**DOI:** 10.3389/fmicb.2013.00116

**Published:** 2013-05-28

**Authors:** Wanpeng Wang, Zongze Shao

**Affiliations:** ^1^State Key Laboratory Breeding Base of Marine Genetic ResourcesXiamen, China; ^2^Key Laboratory of Marine Genetic Resources, Third Institute of Oceanography, State Oceanic AdministrationXiamen, China; ^3^Key Laboratory of Marine Genetic Resources of Fujian ProvinceXiamen, China

**Keywords:** alkane degradation, hydroxylation, monooxygenase, regulations of gene expression, chemotaxis, transporter, AlmA, LadA

## Abstract

Alkanes are major constituents of crude oil. They are also present at low concentrations in diverse non-contaminated because many living organisms produce them as chemo-attractants or as protecting agents against water loss. Alkane degradation is a widespread phenomenon in nature. The numerous microorganisms, both prokaryotic and eukaryotic, capable of utilizing alkanes as a carbon and energy source, have been isolated and characterized. This review summarizes the current knowledge of how bacteria metabolize alkanes aerobically, with a particular emphasis on the oxidation of long-chain alkanes, including factors that are responsible for chemotaxis to alkanes, transport across cell membrane of alkanes, the regulation of alkane degradation gene and initial oxidation.

## INTRODUCTION

Various microorganisms, including bacteria, filamentous fungi and yeasts, can degrade alkanes (van Beilen et al., 2003; Wentzel et al., 2007; Rojo, 2009). Notably, some recently characterized bacterial species are highly specialized for hydrocarbon degradation. These species are called hydrocarbonoclastic bacteria (HCB), and they play a key role in the removal of hydrocarbons from polluted and non-polluted environments (Harayama et al., 2004; Head et al., 2006; Yakimov et al., 2007; Wang et al., 2010a,b).

Of particular importance is *Alcanivorax*, a marine bacterium that can assimilate various linear or branched alkanes but that is unable to metabolize aromatic hydrocarbons, sugars, amino acids, and most other common carbon sources (Liu and Shao, 2005; Yakimov et al., 2007; Wu et al., 2008). *Alcanivorax* bacteria are present in non-polluted seawater in low numbers; however, the number of *Alcanivorax* can increase as a result of an oil spill, and they are believed to play an important role in the natural bioremediation of oil spills worldwide (Kasai et al., 2002; Hara et al., 2003; Harayama et al., 2004; McKew et al., 2007a,b; Yakimov et al., 2007; Wang et al., 2010a,b).

More recently, other HCBs belonging to the genera *Thalassolituus* (Yakimov et al., 2004), *Oleiphilus* (Golyshin et al., 2002), *Oleispira* (Yakimov et al., 2003), *Marinobacter *(Duran, 2010), *Bacillus* and *Geobacillus* (Marchant et al., 2006; Meintanis et al., 2006; Wang et al., 2006) have also been shown to play an important role in the degradation of oil spills in marine environments (Coulon et al., 2007; McKew et al., 2007a,b; Hazen et al., 2010).

Several reviews have covered different aspects of the physiology, enzymes and pathways that are responsible for alkane degradation (van Beilen et al., 2003; van Hamme et al., 2003; Coon, 2005; van Beilen and Funhoff, 2007; Wentzel et al., 2007; Rojo, 2009; Austin and Groves, 2011). This review focuses on recent advances in alkane chemotaxis, across membrane transport and gene regulations. In addition, newly discovered enzymes that are responsible for long-chain alkane mineralization are also discussed.

## CHEMOTAXIS TO LINEAR ALKANES

**FIGURE 1:**
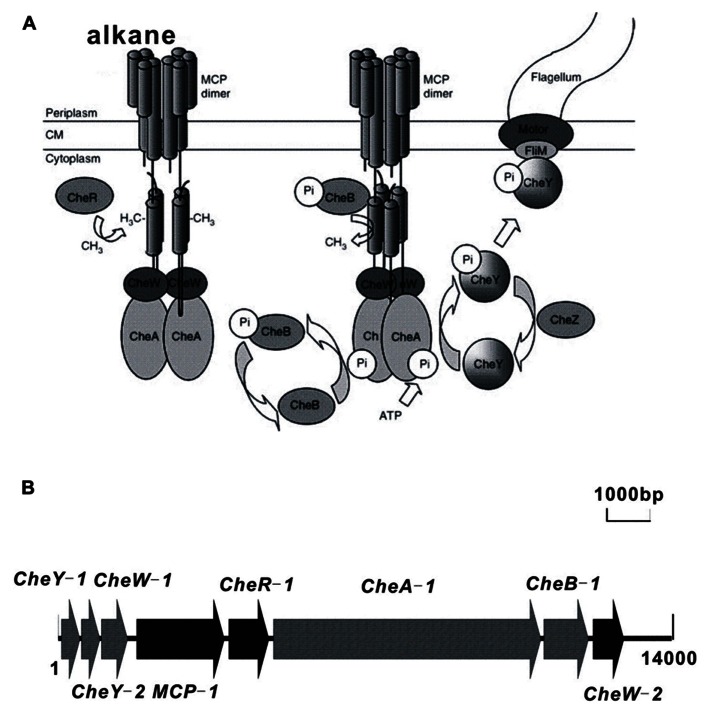
**Schematic diagram of the chemosensory signaling system of *A. dieselolei *B-5. (A)** MCP dimers with associated CheW and CheA proteins are shown in the presence (left) and absence of alkane (right). Cells responding to a gradient of attractant will sense the attractant bound to the periplasmic side of the cognate MCP and will continue swimming in the favorable direction due to the inability of CheA to autophosphorylate. In the absence of CheA-P, CheY remains in the inactive unphosphorylated state, and swimming behavior remains unchanged. Cells swimming down a gradient of attractant will sense the decrease in attractant concentration due to decreased occupancy of the MCPs. Under these conditions, the MCPs undergo a conformational change that is transmitted across the cytoplasmic membrane and stimulates CheA kinase activity. CheA-P phosphorylates CheY, which in its phosphorylated state binds to the FliM protein in the flagellar motor and causes a change in the direction of flagellar rotation allowing the cell to randomly reorient and swim off in a new direction. Dephosphorylation of CheY-P is accelerated by the CheZ phosphatase. Under all conditions, the constitutive methyltransferase CheR methylates specific glutamyl residues on the cytoplasmic side of the MCP. Methylated MCPs stimulate CheA autophosphorylation, thus resetting the system such that further increases in attractant concentration can be detected. The methylesterase, CheB, becomes active when it is phosphorylated by CheA-P. CheB-P competes with CheR and removes methyl groups from the MCPs. CM, cytoplasmic membrane. **(B)** Organization of chemotaxis genes involved in alkane metabolism in* A. dieselolei *B-5. The detailed information of the ORFs of *MCP *gene cluster is presented. MCP, methyl-accepting chemotaxis protein; CheY-1, CheY-like receiver protein; CheY-2, CheY-like receiver protein; CheW-1, CheW-like protein, signal transduction protein; CheW-2, Chemotaxis protein, signal transduction protein; CheA, CheA signal transduction histidine kinase; CheB, CheB methylesterase; CheR, CheR methyltransferase.

Chemotaxis facilitates the movement of microorganisms toward or away from chemical gradients in the environment, and this process plays a role in biodegradation by bringing cells into contact with degradation substrates (Parales and Harwood, 2002; Parales et al., 2008). Alkanes are sources of carbon and energy for many bacterial species and have been shown to function as chemo-attractants for certain microorganisms. A bacterial *Flavimonas oryzihabitans* isolate that was obtained from soil contaminated with gas oil was shown to be chemotactic to gas oil and hexadecane (Lanfranconi et al., 2003). Similarly, *Pseudomonas aeruginosa* PAO1 is chemotactic to hexadecane (Smits et al., 2003). The *tlpS* gene, which is located downstream of the alkane hydroxylase gene *alkB1* in the PAO1 genome, is predicted to encode membrane-bound methyl-accepting chemotaxis proteins (MCP) that may play a role in alkane chemotaxis (Smits et al., 2003), although no experimental evidence exists. Similarly, the gene *alkN* is predicted to encode an MCP that could be involved in alkane chemotaxis in *P. putida *GPo1 (van Beilen et al., 2001). Our recent investigation of the genome sequence of *Alcanivorax dieselolei* B-5 (Lai et al., 2012) identified the alkane chemotaxis machinery of *Alcanivorax*, which consists of eight cytoplasmic chemotaxis proteins that transmit signals from the MCP proteins to the flagellar motors (Figure 1). This chemotaxis machinery is similar to that of *Escherichia coli* (Parales and Ditty, 2010). However, further investigation is necessary to confirm the mechanism of alkane chemotaxis in *A. dieselolei* B-5.

## *n*-ALKANE UPTAKE IN BACTERIA

Although the genes and proteins that enable the passage of aromatic hydrocarbons across the bacterial outer membrane have been identified (van den Berg, 2005; Mooney et al., 2006; Hearn et al., 2008, 2009), the active transport mechanisms involved in alkane uptake remain unclear. Previous reviews(Rojo, 2009) discussed the observation that direct uptake of alkane molecules from the water phase is only possible for low molecular weight alkanes, which are sufficiently soluble to facilitate efficient transport into cells. For medium- and long-chain *n-*alkanes, microorganisms may gain access to these compounds by adhering to hydrocarbon droplets (which is facilitated by the hydrophobic cell surface) or by surfactant-facilitated access, as reviewed by Rojo (2009). Surfactants have been reported to increase the uptake and assimilation of alkanes, such as hexadecane, in liquid culture (Beal and Betts, 2000; Noordman and Janssen, 2002), but their exact role in alkane uptake is not fully understood. Bacteria that are capable of oil degradation usually produce and secrete surfactants of diverse chemical nature that allow alkane emulsification (Yakimov et al., 1998; Peng et al., 2007, 2008; Qiao and Shao, 2010; Shao, 2010). Based on our understanding of biosurfactant structure and the mechanism of outer membrane transport, we speculate that biosurfactants may be excluded from entering the cell and remain in the extracellular milieu.

In *P. putida*, *alkL* in the *alk* operon is postulated to play an important role in alkane transport into the cell (van Beilen et al., 2004; Hearn et al., 2009). Transcriptome analysis of* A. borkumensis* Sk2 revealed that the alkane-induced gene* blc*, encoding the outer membrane lipoprotein Blc, might be involved in alkane uptake because it contains a so-called lipocalin domain (Sabirova et al., 2011). When this domain contacts organic solvents, a small hydrophobic pocket forms and catalyzes the transport of small hydrophobic molecules. More recently, our genome analysis (Lai et al., 2012) and closer examination of *A. dieselolei* B-5indicated that three outer membrane proteins that belong to the long-chain fatty acid transporter protein (FadL) family are involved in alkane transport (unpublished). The FadL homologs are present in many bacteria that are involved in the biodegradation of xenobiotics (van den Berg, 2005), which are usually hydrophobic and probably enter cells by a mechanism similar to that employed for long-chain (LC) fatty acids by FadL in *E. coli*.

## DEGRADATION PATHWAYS OF *n*-ALKANES

The initial terminal hydroxylation of *n-*alkanes can be carried out by enzymes that belong to different families. Microorganisms degrading short-chain length alkanes (C_2_–C_4_, where the subindex indicates the number of carbon atoms of the alkane molecule) have enzymes related to methane monooxygenases (van Beilen and Funhoff, 2007). Strains degrading medium-chain length alkanes (C_5_–C_1__7_) frequently contain soluble cytochrome P450s and integral membrane non-heme iron monooxygenases, such as AlkB (Rojo, 2009; Austin and Groves, 2011).

Interestingly, alkane hydroxylases of long-chain length (LC-) alkanes (>C18) are unrelated to the above alkane hydroxylases as characterized recently. One such hydroxylase, AlmA, is an LC-alkane monooxygenase from* Acinetobacter*. A second hydroxylase is LadA, which is a thermophilic soluble LC-alkane monooxygenase from *Geobacillus* (Feng et al., 2007; Throne-Holst et al., 2007; Wentzel et al., 2007).

The* almA* gene, which encodes a putative monooxygenase belonging to the flavin-binding family, was identified from *Acinetobacter* sp. DSM 17874 (Throne-Holst et al., 2007; Wentzel et al., 2007). This gene encodes the first experimentally confirmed enzyme that is involved in the metabolism of LC *n-*alkanes of C_32_ and longer. We provided the first evidence that the AlmA of the genus *Alcanivorax *functions as an LC-alkane hydroxylase, and found that the gene *almA* in both *A. hongdengensis* A-11-3 and *A. dieselolei* B-5 strains expressed at high levels to facilitate the efficient degradation of LC *n-*alkanes (Liu et al., 2011; Wang and Shao, 2012a). The *almA* gene sequences were present in several bacterial genera capable of LC *n-*alkane degradation, including *Alcanivorax*, *Marinobacter*, *Acinetobacter*, and *Parvibaculum *(Wang and Shao, 2012b). In addition, similar genes are found in other genera in GenBank, such as *Oceanobacter* sp. RED65, *Ralstonia* spp., *Mycobacterium* spp., *Photorhabdus* sp., *Psychrobacter* spp., and *Nocardia farcinica* IFM10152. However, few of these genes have been functionally characterized.

A unique LC-alkane hydroxylase from the thermophilic bacterium *Geobacillus thermodenitrificans* NG80-2 has been characterized. This enzyme is called LadA and oxidizes C_15_–C_36_ alkanes, generating the corresponding primary alcohols (Feng et al., 2007). The LadA crystal structure has been identified, revealing that LadA belongs to the bacterial luciferase family, which is two-component, flavin-dependent oxygenase (Li et al., 2008). LadA is believed to oxidize alkanes by a mechanism similar to that of other flavoprotein monooxygenases, and its ability to recognize and hydroxylate LC-alkanes most likely results from the way in which it captures the alkane(Li et al., 2008). Therefore, the hydroxylases involved in LC-alkane degradation appear to have evolved specifically, which is in contrast with other alkane monooxygenases such as AlkB and P450.

Interestingly, branched-chain alkanes are thought to be more difficult to degrade than linear alkanes (Pirnik et al., 1974). However, *Alcanivorax* bacteria efficiently degrade branched alkanes (Hara et al., 2003). In *A. borkumensis *SK2, isoprenoid hydrocarbon (phytane) strongly induces *P450 *(a) and *alkB2* (Schneiker et al., 2006). In a previous report, we found that both pristane and phytane activate the expression of *alkB1 *and *almA* in* A. dieselolei *B-5 (Liu et al., 2011). In *A. hongdengensis* A-11-3, we recently found that pristane selectively activates the expression of *alkB1*, *P450-3* and *almA *(Wang and Shao, 2012a). However, the metabolic pathways that mediate this activity are poorly understood, although they may involve the ω- or β-oxidation of the hydrocarbon molecule (Watkinson and Morgan, 1990).

## REGULATION OF ALKANE-DEGRADATION PATHWAYS

The expression of the bacterial genes involved in alkane assimilation is tightly regulated. Alkane-responsive regulators ensure that alkane degradation genes are induced only in the presence of the appropriate hydrocarbons. Many microorganisms(Rojo, 2009; Austin and Groves, 2011) contain several sets of alkane degradation systems, each one being active on a particular kind of alkane or being expressed under specific physiological conditions. In these cases, the regulatory mechanisms should assure an appropriate differential expression of each set of enzymes. The regulators that have been characterized belong to different families, including LuxR/MalT, AraC/XylS, and other non-related families (Table 1).

**Table 1:** Transcriptional regulators known or presumed to control the expression of alkane degradation pathways.

Bacterium	Gene	Family	Effector	Evidence	Reference
*P. putida* GPo1	*alkS*	LuxR/MalT	C_6_–C_10_* n-*alkanes	Direct	Sticher et al. (1997) and Panke et al. (1999)
*P. putida* P1	*alkS*	LuxR/MalT	Not tested	Similarity	van Beilen et al. (2001)
*A. borkumensis* SK2	*alkS*	LuxR/MalT	Not tested	Similarity	Schneiker et al. (2006)
*A. borkumensis* SK2	*gntR*	GntR	Not tested	No	Schneiker et al. (2006)
*A. borkumensis* SK2	*araC*	AraC/XylS	Not tested	No	Schneiker et al. (2006)
*A. borkumensis* AP1**	*alkS*	LuxR/MalT	Not tested	Similarity	van Beilen et al. (2004)
*A.hongdengensis* A-11-3	*tetR*	TetR	Not tested	Similarity	Wang and Shao (2012a)
*A.hongdengensis* A-11-3	*gntR*	GntR	Not tested	Similarity	Wang and Shao (2012a)
*A.hongdengensis* A-11-3	*araC1*	AraC/XylS	Not tested	Similarity	Wang and Shao (2012a)
*A.hongdengensis* A-11-3	*araC2*	AraC/XylS	Not tested	Similarity	Wang and Shao (2012a)
*A. dieselolei* B-5	*merR*	MerR	C_14_–C_26_* n*-alkanols	Similarity	Liu et al., (2011)
*A. dieselolei* B-5	*araC1*	AraC/XylS	C_12_–C_26_* n*-alkanols	Similarity	Liu et al., (2011)
*A. dieselolei* B-5	*araC2*	AraC/XylS	C_8_–C_16_* n*-alkanols	Similarity	Liu et al., (2011)
*P. butanovora*	*bmoR*	δ^54^-Dependent	C_2_–C_8_ *n*-alkanols	Direct	Kurth et al. (2008)
*P. aeruginosa* RR1**	*gntR*	GntR	C_10_-C_20_ *n*-alkanols	Indirect	Marιn et al. (2003)
*Acinetobacter* sp. ADP1**	*alkR*	AraC/XylS	C_7_-C_18_* n*-alkanols	Direct	Ratajczak et al. (1998)
*Acinetobacter* sp. M1**	*alkRa*	AraC/XylS	>C_22_* n*-alkanols	Indirect	Tani et al. (2001)
*Acinetobacter* sp. M1**	*alkRb*	OruR	C_16_–C_22_* n*-alkanols	Indirect	Tani et al. (2001)

### REGULATION OF THE ALKANE DEGRADATION PATHWAY IN *Pseudomonas* spp.

*Pseudomonas butanovora* species oxidize C_2_–C_8_ *n-*alkanes into the corresponding alcohols with an alkane monooxygenase termed butane monooxygenase (BMO). BMO is a multimeric protein that is formed by the products of the* bmoXYBZDC* operon (Sluis et al., 2002). The expression of the genes encoding BMO is activated by BmoR, a δ^54^-dependent transcriptional regulator that recognizes alcohols and aldehydes derived from the C_2_–C_8_ *n-*alkanes that are substrates of BMO, although BmoR does not recognize the alkanes themselves (Kurth et al., 2008).

In *P. putida *GPo1, the OCT plasmid encodes all of the genes required for the assimilation of C_3_–C_13_ alkanes (van Beilen et al., 1994, 2005; Johnson and Hyman, 2006). The genes in this pathway are grouped into two clusters, *alkBFGHJKL* and *alkST* (van Beilen et al., 1994, 2001). The *alkBFGHJKL *operon is transcribed from a promoter named *PalkB*, whose expression requires the transcriptional activator AlkS and the presence of alkanes (Kok et al., 1989; Panke et al., 1999). An AlkS-dependent reporter system based on a *PalkB-luxAB* fusion showed that C_5_–C_10_ alkanes are efficient activators of the AlkS regulator (Sticher et al., 1997). When alkanes become available, AlkS binds and represses *PalkS1* more efficiently than it does in the absence of alkanes. From this binding site, AlkS activates the *PalkS2* promoter, resulting in high expression of the *alkST* genes (Canosa et al., 2000). Therefore, this pathway is controlled by a positive feedback mechanism that is driven by AlkS.

### REGULATION OF THE ALKANE DEGRADATION PATHWAY IN *Alcanivorax* spp.

A gene similar to *alkS* in *P. putida *GPo1 is located upstream of *alkB1* in *A. borkumensis* SK2, and AlkS is predicted to be an alkane-responsive transcriptional activator. The expression level of AlkS in strain SK2 cells grown in hexadecane is higher than that of pyruvate-grown cells (Sabirova et al., 2006). Evidence suggests that in *A. borkumensis*, AlkS activates the expression of *alkB1*, a gene that encodes an alkane hydroxylase, in response to alkanes. However, it is unlikely that AlkS regulates the expression of *alkB2*, despite the induction of this gene in response to alkanes (van Beilen et al., 2004). Interestingly, a gene encoding a transcriptional regulator belonging to the GntR family is located immediately upstream of *alkB2*; however, its role in *alkB2* expression has not been reported. *A. borkumensis* has three genes encoding cytochrome P450 of the CYP153 family (Schneiker et al., 2006). A gene encoding a transcriptional regulator belonging to the AraC family is located close to *P450-1*, but its role in regulating the *P450-1* gene cluster has not been investigated (Schneiker et al., 2006).

In* A. hongdengensis, *a gene downstream of *alkB1 *encodes a protein that is similar to TetR family transcriptional regulators (Wang and Shao, 2012a). In addition, a gene encoding a transcriptional regulator belonging to the GntR family is located just upstream of *alkB2*, although its role in the regulation of *alkB2* is not known (Wang and Shao, 2012a). Genes encoding transcriptional regulators belonging to the AraC family are located near *P450-1* and *P450-2* (Wang and Shao, 2012a). Similar to many of the genes described above, their role in the regulation of the corresponding P450 genes requires further investigation.

Three regulators that are involved in alkane degradation were identified in the* A. dieselolei* strain B-5 genome sequence, and they belong to different MerR and AraC families (Table 1). Regulatory genes are located upstream of *alkB1 *and* P450*, and the proteins encoded by these genes are 46 and 64% similar to MerR and AraC from *P. aeruginosa *and *A. borkumensis* SK2, respectively (Liu et al., 2011). Downstream of *alkB2*, there is a gene encoding a transcriptional regulator that shares 61% similarity with AraC from* Marinobacter* sp. ELB17 (Liu et al., 2011). Therefore, *Alcanivorax *strains usually encode multiple alkane hydroxylases that are expressed under the control of different regulators encoded in the same gene cluster as the monooxygenase gene. Our lab is using strain B-5 as a model system to study how cells modulate the expression of these genes in response to different alkanes with varied chain lengths.

### GLOBAL REGULATION OF THE ALKANE DEGRADATION PATHWAY

The expression of alkane degradation pathway genes is often down regulated by complex global regulatory controls that ensure that the genes are expressed only under the appropriate physiological conditions or in the absence of any preferred compounds (Rojo, 2009). Two global regulatory networks exist. One network relies on the global regulatory protein Crc (Yuste and Rojo, 2001), while the other network receives information from cytochrome o ubiquinol oxidase (Cyo), which is a component of the electron transport chain (Dinamarca et al., 2002, 2003).

The Crc is an RNA-binding protein that interacts with the 5′ end of the *alkS *mRNA, inhibiting translation (Moreno et al., 2007). A recent study further showed that Crc inhibits the induction of the alkane degradation pathway by limiting not only the translation of their transcriptional activators but also that of genes involved in the entire alkane degradation pathway in* P. putida* (Hernández-Arranz et al., 2013). In addition, results of this study suggests that Crc follows a multi-step strategy in many cases, targeting uptake, transcription regulation, and/or the production of the associated pathways’ catabolic enzymes (Hernández-Arranz et al., 2013).

However, when cells grow in a minimal salt medium containing succinate as the carbon source, the activity of Crc is low; instead, Cyo terminal oxidase play a key role in the global control that inhibits the induction of the alkane degradation genes (Yuste and Rojo, 2001; Dinamarca et al., 2003). Cyo is one of the five terminal oxidases that have been characterized in* P. putida*. Inactivation of the Cyo terminal oxidase partially relieves the repression exerted on the alkane degradation pathway under several conditions, while inactivation of any of the other four terminal oxidases does not (Dinamarca et al., 2002; Morales et al., 2006). Cyo affects the expression of many other genes, and this enzyme has been proposed to be a component of a global regulatory network that transmits information regarding the activity of the electron transport chain to coordinate respiration and carbon metabolism (Petruschka et al., 2001; Morales et al., 2006). The expression of the *cyo* genes encoding the subunits of Cyo terminal oxidase varies depending on oxygen levels and carbon source, and there is a clear correlation between Cyo levels and the extent of alkane degradation pathway repression (Dinamarca et al., 2003).

## CONCLUDING REMARKS

Research in the last few years has resulted in many new insights into the mechanism of alkane degradation by microorganisms, including the upstream regulations and the long-chain length alkane oxidation. Investigations using “omics” strategies will help us to better understand the global metabolic networks within a microbial cell and the overall process of bacterial alkane-dependent chemotaxis, alkane transport, gene expression regulation and complete mineralization.

## Conflict of Interest Statement

The authors declare that the research was conducted in the absence of any commercial or financial relationships that could be construed as a potential conflict of interest.
